# Research on the simplified calculation method of non-limit active earth pressure of limited soil mass in adjacent foundation pits

**DOI:** 10.1371/journal.pone.0349800

**Published:** 2026-06-24

**Authors:** Zhenbo Zhang, Zhengyuan Wang, Fei Xu, Fei Wu, Zhichun Liu, Xiaofan Chen, Jingliang Kang

**Affiliations:** 1 Institute of Health Diagnosis and Control of Large Structures, Shijiazhuang Tiedao University, Shijiazhuang, Hebei, China; 2 Yanzhao Modern Transportation Laboratory, Shijiazhuang, Hebei, China; 3 School of Civil Engineering, Shijiazhuang Tiedao University, Shijiazhuang, Hebei, China; 4 School of Traffic and Transportation, Shijiazhuang Tiedao University, Shijiazhuang, Hebei, China; 5 Beijing Rail and Transit Design & Research Institute Co., Ltd., Beijing, China; 6 China Railway Design Group Co., Ltd., Tianjin, China; China Construction Fourth Engineering Division Corp. Ltd, CHINA

## Abstract

This study aims to solve two critical issues in close-proximity foundation pit engineering: the cumbersome calculation process of non-limit active earth pressure for confined soil masses, and the unclear quantitative relationship between earth pressure magnitude and retaining structure displacement. By establishing the coupling relationship among static earth pressure, limit active earth pressure and non-limit active earth pressure, this paper derives analytical expressions for both the intensity and resultant force of non-limit active earth pressure corresponding to five distinct soil failure modes. The proposed theoretical model is validated through systematic laboratory model tests and finite element numerical simulations. Furthermore, the concept of non-limit active earth pressure isograms is introduced, and the influence mechanisms of various engineering parameters on the isogram distribution characteristics are quantitatively analyzed, revealing their inherent nonlinear distribution laws. Finally, a practical simplified calculation method is proposed, which provides a reliable theoretical basis and technical reference for the design and construction of retaining structures in close-proximity foundation pit engineering.

## 1. Introduction

With the rapid development of urban underground space utilization, an increasing number of foundation pit projects are being constructed in extremely close proximity to existing underground structures, forming a confined soil zone between the new excavation and the adjacent structures. The stress state, strain field and earth pressure distribution within this limited soil mass are highly dependent on the lateral deformation of the retaining structure. If the classical Coulomb [1] and Rankine [2] earth pressure theories, which are based on the semi-infinite soil assumption, are directly applied to such conditions, the calculated results will significantly overestimate the actual earth pressure acting on the retaining structure, leading to unnecessary material waste and increased engineering cost [3]. Therefore, in-depth research on the non-limit active earth pressure of limited soil masses is of great theoretical significance and engineering practical value.

In recent decades, extensive research has been conducted on the non-limit earth pressure problem of confined soils, which can be broadly categorized into three main approaches: theoretical derivation, laboratory testing and numerical simulation. In terms of theoretical research, scholars have made significant progress by improving traditional earth pressure theories. Wang et al. [4], Chen et al. [5] and Tang et al. [6] established basic calculation frameworks for non-limit earth pressure of limited soil masses, and systematically revealed the influence laws of key parameters on earth pressure distribution. Jiang et al. [7] further introduced the soil arching effect into the calculation model, and derived analytical solutions for non-limit active earth pressure applicable to both cohesive and cohesionless soils. Ma et al. [8] proposed a novel calculation method by incorporating interlayer shear stress, which overcomes the limitation that traditional models cannot obtain explicit integral solutions. Ghobadi et al. [9] combined the principal stress rotation method with a bilinear slip surface assumption to derive an analytical solution for active earth pressure in narrow backfills. It should be noted that all the above theoretical studies were conducted under the translational displacement mode of retaining walls. In addition, Xia et al. [10] extended the research scope to passive earth pressure, and investigated its distribution characteristics under three typical displacement modes of retaining walls through theoretical analysis.

Laboratory testing is an indispensable means to verify theoretical models and reveal soil failure mechanisms. Hu et al. [11,12] adopted advanced Particle Image Velocimetry (PIV) technology to establish a calculation procedure for non-limit passive earth pressure suitable for flexible retaining structures. They also systematically analyzed the failure surface morphology of limited soil masses and the earth pressure distribution law under the wall-bottom rotation mode. Frydman et al. [13], Take et al. [14], Yang et al. [15] and Rui et al. [16] carried out a series of model tests to investigate the effects of backfill width and wall height ratio on earth pressure under the translational mode. Zhang et al. [17] combined theoretical analysis with model tests to propose a three-stage slip surface model for foundation pits adjacent to existing metro stations, and quantitatively analyzed the influences of limited soil width and soil internal friction angle on earth pressure.

Numerical simulation has become a powerful tool for studying complex geotechnical problems due to its ability to simulate various working conditions and reveal internal stress-strain fields. Lin et al. [18], Fan et al. [19], Potts et al. [20] and Benmeddour et al. [21] used numerical simulation software to analyze the influences of limited soil bottom width and internal friction angle on earth pressure distribution and failure surface morphology under different displacement modes of retaining walls. Fathipour et al. [22] employed the Finite Element Limit Analysis (FELA) method to study unsaturated soil earth pressure, and examined the effects of groundwater level and seepage on earth pressure distribution.

Although significant progress has been made in this field, existing calculation methods for non-limit active earth pressure of limited soil masses are generally too complex for practical engineering applications. Moreover, the quantitative relationship between non-limit active earth pressure and retaining structure displacement in confined soil conditions has not been fully clarified. To address these gaps, this paper proposes a displacement-dependent calculation method for non-limit active earth pressure of limited soil masses based on the relationship among static, limit active and non-limit active earth pressures. Analytical formulas for non-limit active earth pressure resultant force corresponding to five soil failure modes are derived. The correctness and rationality of the proposed method are verified through laboratory model tests and finite element numerical simulations. On this basis, the concept of non-limit active earth pressure isograms is introduced, and their calculation process and parameter influence laws are systematically analyzed. Finally, a practical simplified calculation method is presented, along with recommended values of the spatial position relationship coefficient, which provides a convenient tool for engineering designers.

## 2. Theoretical calculation methods for non-limit active earth pressure

### 2.1 Basic calculation principle

**Fig 1:**
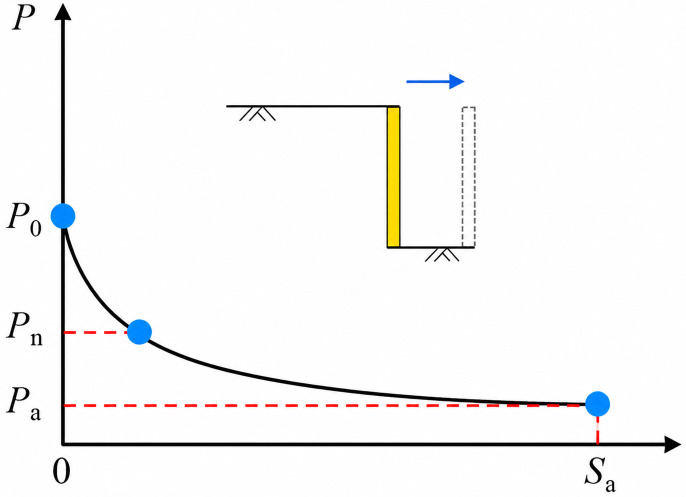
Relationship between earth pressure and retaining wall displacement.

The earth pressure decreases as the displacement of the retaining wall (*s*) increases. When the displacement is 0, the earth pressure corresponds to the static earth pressure (*P*_0_). As the displacement increases from 0 to *S*_*a*_, the earth pressure represents the non-limit active earth pressure (*P*_*n*_). At the displacement of *S*_*a*_, where the soil mass reaches the limit state, the earth pressure is referred to as the limit active earth pressure (*P*_*a*_). The relationship among these three states is illustrated in Fig 1.

Based on the above-mentioned variation law and drawing on the relevant research results of Zhang et al. [23], this paper proposes a displacement-dependent non-limit active earth pressure calculation formula using a Sigmoid function to describe the variation of the earth pressure coefficient, as shown in Eq. (1).


$$\begin{array}{c} \left\{ \begin{array}{c} @l@p_{\text{n}}=p_{\text{0}}+k_{\text{n}}\left(p_{\text{a}}-p_{\text{0}}\right)\\ k_{\text{n}}=\frac{\left[1-e^{c\left(\frac{s}{s_{\text{a}}}\right)}\right]\left(1+e^{c}\right)}{\left[1+e^{c\left(\frac{s}{s_{\text{a}}}\right)}\right]\left(1-e^{c}\right)}\\ c=2.5ln\left[\frac{\text{3}}{e^{{tan}^{\text{2}}\left(\pi /4-\varphi /2\right)}-1}+1\right] \end{array}\right. \end{array}$$ (1)

In equation (1), *p*_*n*_ is the non-limit active earth pressure; *p*_*0*_ is the static earth pressure, where *p*_0_=(1-sin*φ*)*γz*; *p*_*a*_ is the limit active earth pressure, calculated using the limit active earth pressure formula proposed by Zhang et al. [23]; *k*_*n*_ is the non-limit active earth pressure coefficient, *s* is the displacement of the retaining wall, *s*_*a*_ is the displacement required for the soil to reach the active limit state, *φ* is the internal friction angle of the soil, and *c* is a function related to the internal friction angle of the soil.

### 2.2 Non-limit active earth pressure intensity

**Fig 2:**
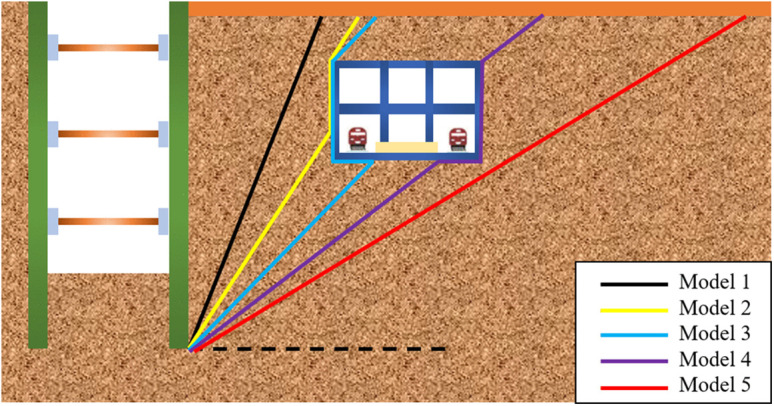
Five failure modes of soil mass.

According to the relative positional relationship between the potential slip surface of foundation pit soil and the adjacent existing underground structures, the failure modes of limited soil masses can be divided into five categories [23], as illustrated in Fig 2.

By substituting the limit active earth pressure intensity formula and static earth pressure formula obtained in the study of Zhang et al. [23], as well as the non-limit active earth pressure coefficient (*k*_*n*_) proposed in this paper, into equation (1), the non-limit active earth pressure intensity formulas for the five failure modes of soil mass are obtained, as shown in equations (2)–(12) respectively.

The non-limit active earth pressure intensity formula for the first soil failure mode is


$$\begin{array}{c} p_{\text{n}}=\left(1-k_{\text{n}}\right)\left(1-sin\varphi \right)\gamma z+k_{\text{n}}k_{\text{a}}m(H+D-z{)}^{a_{\text{1}}}+k_{\text{n}}k_{\text{a}}\frac{\gamma (H+D-z)}{a_{\text{1}}-1} \end{array}$$ (2)

In equation (2), *a*_1_ and *m* are respectively


$$\begin{array}{c} a_{\text{1}}=k_{\text{a}}tan\delta_{\text{1}}tan\theta +\frac{k_{\text{a}}{tan}^{\text{2}}\theta tan\varphi }{\left(tan\theta \text{-}tan\varphi \right)}+\frac{k_{\text{a}}tan\theta }{\left(tan\theta \text{-}tan\varphi \right)}-1 \end{array}$$ (3)

$$\begin{array}{c} m\text{=}\left[q-\frac{\gamma (H+D)}{a_{\text{1}}-1}\right](H+D{)}^{-a_{\text{1}}} \end{array}$$ (4)

The formula for the non-limit active earth pressure intensity of the second soil failure mode is


$$\begin{array}{c} p_{\text{n}}\text{=}\left\{ \begin{array}{c} @l@\left(1-k_{\text{n}}\right)\left(1-sin\varphi \right)\gamma z+k_{\text{n}}k_{\text{a}}\left[m_{\text{1}}(btan\theta +h_{j}-z{)}^{a_{\text{1}}}+\frac{\gamma (btan\theta +h_{j}-z)}{a_{\text{1}}-1}\right]\begin{array}{cc} & 0\leq z\leq h_{\text{j}} \end{array}\\ \left(1-k_{\text{n}}\right)\left(1-sin\varphi \right)\gamma z+k_{\text{n}}k_{\text{a}}\left[m_{\text{2}}e^{-a_{\text{2}}z}+\frac{\gamma }{a_{\text{2}}}\right]\begin{array}{ccccccccc} & & & & & & & & h_{\text{j}}\leq z\leq H+D-btan\theta \end{array}\\ \left(1-k_{\text{n}}\right)\left(1-sin\varphi \right)\gamma z+k_{\text{n}}k_{\text{a}}\left[m_{\text{3}}(H+D-z{)}^{a_{\text{1}}}+\frac{\gamma (H+D-z)}{a_{\text{1}}-1}\right]\begin{array}{cccc} & & & H+D-btan\theta \leq z\leq H+D \end{array} \end{array}\right. \end{array}$$ (5)

In equation (5), *m*_1_, *m*_2_, *m*_3_ and *a*_2_ are respectively


$$\begin{array}{c} \left\{ \begin{array}{c} @l@m_{\text{1}}\text{=}\left[q-\frac{\gamma \left(btan\theta +h_{j}\right)}{a_{\text{1}}-1}\right]{\left(btan\theta +h_{j}\right)}^{-a_{\text{1}}}\\ m_{\text{2}}\text{=}\left\{\left[m_{\text{1}}{\left(btan\theta \right)}^{a_{\text{1}}}+\frac{\gamma btan\theta }{a_{\text{1}}-1}\right]-\frac{\gamma }{a_{\text{2}}}\right\}e^{a_{\text{2}}h_{j}}\\ m_{\text{3}}\text{=}\frac{m_{\text{2}}e^{-a_{\text{2}}\left(D+H-btan\theta \right)}+\frac{\gamma }{a_{\text{2}}}-\frac{\gamma btan\theta }{a_{\text{1}}-1}}{{\left(btan\theta \right)}^{a_{\text{1}}}}\\ a_{\text{2}}=\frac{k_{\text{a}}}{b}\left(tan\delta_{\text{1}}+tan\delta_{\text{2}}\right) \end{array}\right. \end{array}$$ (6)

The formula for the non-limit active earth pressure intensity of the third soil failure mode is


$$\begin{array}{c} p_{\text{n}}=\left\{ \begin{array}{c} @l@\left(1-k_{\text{n}}\right)\left(1-sin\varphi \right)\gamma z+k_{\text{n}}k_{\text{a}}\left[m_{\text{1}}(btan\theta +h_{j}-z{)}^{a_{\text{1}}}+\frac{\gamma (btan\theta +h_{j}-z)}{a_{\text{1}}-1}\right]\begin{array}{cc} & 0\leq z\leq h_{\text{j}} \end{array}\\ \left(1-k_{\text{n}}\right)\left(1-sin\varphi \right)\gamma z+k_{\text{n}}k_{\text{a}}(m_{\text{2}}e^{-a_{\text{2}}z}+\frac{\gamma }{a_{\text{2}}})\begin{array}{ccccccccc} & & & & & & & & h_{\text{j}}\leq z\leq h_{\text{j}}+h_{\text{0}} \end{array}\\ \left(1-k_{\text{n}}\right)\left(1-sin\varphi \right)\gamma z+k_{\text{n}}k_{\text{a}}\left[m_{\text{4}}(H+D-z{)}^{a_{\text{1}}}+\frac{\gamma (H+D-z)}{a_{\text{1}}-1}\right]\begin{array}{ccc} & & h_{\text{j}}+h_{\text{0}}\leq z\leq H+D \end{array} \end{array}\right. \end{array}$$ (7)

In equation (7), *m*_4_ is


$$\begin{array}{c} m_{\text{4}}\text{=}\frac{\left(m_{\text{2}}e^{-a_{\text{2}}(h_{\text{0}}+h_{j})}+\frac{\gamma }{a_{\text{2}}}\right)\frac{btan\theta }{h_{s}}-\frac{\gamma (H+D-h_{\text{0}}-h_{j})}{a_{\text{1}}-1}}{(H+D-h_{\text{0}}-h_{j}{)}^{a_{\text{1}}}} \end{array}$$ (8)

The formula for the non-limit active earth pressure intensity of the fourth soil failure mode is


$$\begin{array}{c} p_{\text{n}}=\left\{ \begin{array}{c} @l@\begin{array}{cc} \left(1-k_{\text{n}}\right)\left(1-sin\varphi \right)\gamma z+k_{\text{n}}k_{\text{a}}\left[m_{\text{5}}[(b+b_{\text{0}})tan\theta +h_{\text{0}}+h_{j}-z{]}^{a}+\frac{\gamma [(b+b_{\text{0}})tan\theta +h_{\text{0}}+h_{j}-z]}{a-1}\right] & 0\leq z\leq h_{\text{j}} \end{array}\\ \begin{array}{cc} \left(1-k_{\text{n}}\right)\left(1-sin\varphi \right)\gamma z+k_{\text{n}}k_{\text{a}}\left[m_{\text{6}}{\left[\left(b+b_{\text{0}}\right)tan\theta +h_{\text{0}}+h_{j}-z\right]}^{a}+\frac{\gamma_{\text{D}}\left[\left(b+b_{\text{0}}\right)tan\theta +h_{\text{0}}+h_{j}-z\right]}{a-1}\right] & h_{\text{j}}\leq z\leq h_{\text{j}}+h_{\text{0}} \end{array}\\ \begin{array}{cccc} \left(1-k_{\text{n}}\right)\left(1-sin\varphi \right)\gamma z+k_{\text{n}}k_{\text{a}}\left[m_{\text{7}}(H+D-z{)}^{a}+\frac{\gamma (H+D-z)}{a-1}\right] & h_{\text{j}}+h_{\text{0}}\leq z\leq H+D & & \end{array} \end{array}\right. \end{array}$$ (9)

In equation (9), *m*_5_, *m*_6_, *m*_7_, *a*, and *γ*_*D*_ are respectively


$$\begin{array}{c} \left\{ \begin{array}{c} @l@\gamma_{\text{D}}\text{=}\frac{G_{\text{0}}\text{+}\gamma \left(bh_{\text{0}}\text{+}\frac{\overset{\text{2}}{\underset{\text{0}}{h}}}{2tan\theta }\right)}{b_{\text{0}}h_{\text{0}}+bh_{\text{0}}\text{+}\frac{\overset{\text{2}}{\underset{\text{0}}{h}}}{2tan\theta }}=\frac{2tan\theta G_{\text{0}}\text{+}\gamma \left(2tan\theta bh_{\text{0}}\text{+}\overset{\text{2}}{\underset{\text{0}}{h}}\right)}{2tan\theta b_{\text{0}}h_{\text{0}}+2tan\theta bh_{\text{0}}\text{+}\overset{\text{2}}{\underset{\text{0}}{h}}}\\ m_{\text{5}}\text{=}\left[q-\frac{\gamma [(b+b_{\text{0}})tan\theta +h_{\text{0}}+h_{j}]}{a-1}\right][(b+b_{\text{0}})tan\theta +h_{\text{0}}+h_{j}{]}^{-a}\\ m_{\text{6}}\text{=}\left[m_{\text{5}}[(b+b_{\text{0}})tan\theta +h_{\text{0}}{]}^{a}+\frac{\left(\gamma -\gamma_{\text{D}})[(b+b_{\text{0}})tan\theta +h_{\text{0}}\right]}{a-1}\right]{\left[\left(b+b_{\text{0}}\right)tan\theta +h_{\text{0}}\right]}^{-a}\\ m_{\text{7}}={h_{s}}^{-a}\left[\left[m_{\text{6}}{\left(btan\theta +b_{\text{0}}tan\theta \right)}^{a}+\frac{\gamma_{\text{D}}(b+b_{\text{0}})tan\theta }{a-1}\right]\frac{(b+b_{\text{0}})tan\theta }{h_{s}}-\frac{\gamma h_{s}}{a-1}\right]\\ a=\frac{k_{\text{a}}tan\theta }{tan\theta -tan\varphi } \end{array}\right. \end{array}$$ (10)

The formula for the non-limit active earth pressure intensity of the fifth soil failure mode is


$$\begin{array}{c} p_{\text{n}}=\left\{ \begin{array}{c} @l@\begin{array}{cc} \left(1-k_{\text{n}}\right)\left(1-sin\varphi \right)\gamma z+k_{\text{n}}k_{\text{a}}\left[m_{\text{5}}[\left(b+b_{\text{0}}\right)tan\theta +h_{\text{0}}+h_{j}-d{]}^{a}+\frac{\gamma \left[\left(b+b_{\text{0}}\right)tan\theta +h_{\text{0}}+h_{j}-z\right]}{a-1}\right] & 0\leq z\leq h_{\text{j}} \end{array}\\ \begin{array}{ccc} \left(1-k_{\text{n}}\right)\left(1-sin\varphi \right)\gamma z+ & k_{\text{n}}k_{\text{a}}\left[m_{\text{8}}(H+D-z{)}^{a}+\frac{\gamma_{\text{D1}}(H+D-z)}{a-1}\right] & h_{\text{j}}\leq z\leq h_{\text{j}}+h_{\text{0}} \end{array}\\ \begin{array}{ccc} \left(1-k_{\text{n}}\right)\left(1-sin\varphi \right)\gamma z+k_{\text{n}}k_{\text{a}}\left[m_{\text{9}}(H+D-z{)}^{a}+\frac{\gamma (H+D-z)}{a-1}\right] & & h_{\text{j}}+h_{\text{0}}\leq z\leq H+D \end{array} \end{array}\right. \end{array}$$ (11)

In equation (11), *m*_8_ and *m*_9_ are respectively


$$\begin{array}{c} \left\{ \begin{array}{c} @l@m_{\text{8}}\text{=}\left[m(h_{\text{0}}+h_{s}{)}^{a}+\frac{\left(\gamma -\gamma_{\text{D1}})(h_{\text{0}}+h_{s}\right)}{a-1}\right](h_{\text{0}}+h_{s}{)}^{-a}\\ m_{\text{9}}\text{=}\left[m_{\text{8}}{h_{s}}^{a}+\frac{(\gamma_{\text{D1}}-\gamma )h_{s}}{a-1}\right]{h_{s}}^{-a}\\ \gamma_{\text{D1}}\text{=}\frac{2tan\theta G_{\text{0}}\text{+}\gamma h_{\text{0}}(2h_{\text{s}}+h_{\text{0}}-2b_{\text{0}}tan\theta )}{2h_{\text{0}}h_{\text{s}}+\overset{\text{2}}{\underset{\text{0}}{h}}} \end{array}\right. \end{array}$$ (12)

In equations (2)–(12), *k*_*a*_ is the Rankine active earth pressure coefficient, *k*_*a*_ = tan^2^(π/4-*φ*/2); *δ* is the wall-soil friction angle, taken as 2*φ*/3; *θ* is the inclination angle of the potential soil slip surface, taken as *θ* = *π*/4 + *φ*/2; *q* is the surcharge; *φ* is the soil internal friction angle; *δ*_2_ is the friction angle between the soil and the existing underground structure, taken as 2*φ*/3; The self-weight of the metro station is *G*_0_.

### 2.3 Non-limit active earth pressure resultant force

Integrating the non-limit active earth pressure intensity formulas for the five soil failure modes yields the non-limit active earth pressure resultant force formulas, as shown in equations (13)–(17) respectively.

The resultant force formula for the first soil failure mode is


$$\begin{array}{c} \begin{array}{c} E_{\text{na}}=\int_{\text{0}}^{H+D}p_{\text{n}}\text{d}z=\left(1-k_{\text{n}}\right)\int_{\text{0}}^{H+D}p_{\text{0}}\text{d}z+k_{\text{n}}\int_{\text{0}}^{H+D}p_{\text{a}}\text{d}z\\ =\left(1-k_{\text{n}}\right)\left(1-sin\varphi \right)\gamma \int_{\text{0}}^{H+D}z\text{d}z+k_{\text{n}}\int_{\text{0}}^{H+D}k_{\text{a}}\left[m(H+D-z{)}^{a_{\text{1}}}+\frac{\gamma (H+D-z)}{a_{\text{1}}-1}\right]\text{d}z\\ =\left(1-k_{\text{n}}\right)\left(1-sin\varphi \right)\frac{\gamma }{\text{2}}{\left(H+D\right)}^{\text{2}}+k_{\text{n}}\left[\frac{k_{\text{a}}\gamma {\left(H+D\right)}^{\text{2}}}{2\left(a_{\text{1}}-1\right)}+\frac{k_{\text{a}}m{\left(H+D\right)}^{a_{\text{1}}+1}}{a_{\text{1}}+1}\right] \end{array} \end{array}$$ (13)

The resultant force formula for the second soil failure mode is


$$\begin{array}{c} \begin{array}{c} E_{\text{na}}=\int_{\text{0}}^{H+D}p_{\text{n}}\text{d}z=\left(1-k_{\text{n}}\right)\int_{\text{0}}^{H+D}p_{\text{0}}\text{d}z+k_{\text{n}}\int_{\text{0}}^{H+D}p_{\text{a}}\text{d}z\\ =\left(1-k_{\text{n}}\right)\left(1-sin\varphi \right)\frac{\gamma }{\text{2}}{\left(H+D\right)}^{\text{2}}+k_{\text{n}}\left[\frac{k_{\text{a}}\gamma {\left(btan\theta +h_{\text{j}}\right)}^{\text{2}}}{2\left(a_{\text{1}}-1\right)}\right]\\ +k_{\text{n}}k_{\text{a}}\left[\frac{m_{\text{1}}{\left(btan\theta +h_{\text{j}}\right)}^{a_{\text{1}}+1}+\left(m_{\text{3}}-m_{\text{1}}\right){\left(btan\theta \right)}^{a_{\text{1}}+1}}{a_{\text{1}}+1}\right]\\ +k_{\text{n}}k_{\text{a}}\left[\frac{\gamma \left(h_{\text{0}}+h_{s}-btan\theta \right)+m_{\text{2}}\left(e^{-a_{\text{2}}h_{\text{j}}}-e^{-a_{\text{2}}\left(H+D-btan\theta \right)}\right)}{a_{\text{2}}}\right] \end{array} \end{array}$$ (14)

The resultant force formula for the third soil failure mode is


$$\begin{array}{c} \begin{array}{c} E_{\text{na}}=\int_{\text{0}}^{H+D}p_{\text{n}}\text{d}z=\left(1-k_{\text{n}}\right)\int_{\text{0}}^{H+D}p_{\text{0}}\text{d}z+k_{\text{n}}\int_{\text{0}}^{H+D}p_{\text{a}}\text{d}z\\ =\left(1-k_{\text{n}}\right)\left(1-sin\varphi \right)\frac{\gamma }{\text{2}}{\left(H+D\right)}^{\text{2}}+k_{\text{n}}k_{\text{a}}\frac{\gamma \left[{\left(btan\theta +h_{\text{j}}\right)}^{\text{2}}+\overset{\text{2}}{\underset{s}{h}}-{\left(btan\theta \right)}^{\text{2}}\right]}{2\left(a_{\text{1}}-1\right)}\\ +k_{\text{n}}k_{\text{a}}\frac{m_{\text{1}}\left[{\left(btan\theta +h_{\text{j}}\right)}^{a_{\text{1}}+1}-{\left(btan\theta \right)}^{a_{\text{1}}+1}\right]+m_{\text{4}}\overset{a_{\text{1}}+1}{\underset{s}{h}}}{a_{\text{1}}+1}+k_{\text{n}}k_{\text{a}}\frac{\gamma h_{\text{0}}+m_{\text{2}}\left[e^{-a_{\text{2}}h_{\text{j}}}-e^{-a_{\text{2}}\left(h_{\text{j}}+h_{\text{0}}\right)}\right]}{a_{\text{2}}} \end{array} \end{array}$$ (15)

The resultant force formula for the fourth soil failure mode is


$$\begin{array}{c} \begin{array}{c} E_{\text{na}}=\int_{\text{0}}^{H+D}p_{\text{n}}\text{d}z=\left(1-k_{\text{n}}\right)\int_{\text{0}}^{H+D}p_{\text{0}}\text{d}z+k_{\text{n}}\int_{\text{0}}^{H+D}p_{\text{a}}\text{d}z\\ =\left(1-k_{\text{n}}\right)\left(1-sin\varphi \right)\frac{\gamma }{\text{2}}{\left(H+D\right)}^{\text{2}}\\ +k_{\text{n}}k_{\text{a}}\frac{\gamma \overset{\text{2}}{\underset{s}{h}}+\gamma {\left[h_{\text{j}}+h_{\text{0}}+\left(b+b_{\text{0}}\right)tan\theta \right]}^{\text{2}}-\left(\gamma -\gamma_{\text{D}}\right){\left[h_{\text{0}}+\left(b+b_{\text{0}}\right)tan\theta \right]}^{\text{2}}-\gamma_{\text{D}}{\left[\left(b+b_{\text{0}}\right)tan\theta \right]}^{\text{2}}}{2\left(a-1\right)}\\ +k_{\text{n}}k_{\text{a}}\frac{m_{\text{5}}{\left[h_{\text{j}}+h_{\text{0}}+\left(b+b_{\text{0}}\right)tan\theta \right]}^{a+1}-m_{\text{6}}{\left[\left(b+b_{\text{0}}\right)tan\theta \right]}^{a+1}}{a+1}\\ +k_{\text{n}}k_{\text{a}}\frac{\left(m_{\text{6}}-m_{\text{5}}\right){\left[h_{\text{0}}+\left(b+b_{\text{0}}\right)tan\theta \right]}^{a+1}+m_{\text{7}}\overset{a+1}{\underset{s}{h}}}{a+1} \end{array} \end{array}$$ (16)

The resultant force formula for the fifth soil failure mode is


$$\begin{array}{c} \begin{array}{c} E_{\text{na}}=\int_{\text{0}}^{H+D}p_{\text{n}}dz=\left(1-k_{\text{n}}\right)\int_{\text{0}}^{H+D}p_{\text{0}}dz+k_{\text{n}}\int_{\text{0}}^{H+D}p_{\text{a}}dz\\ =\left(1-k_{\text{n}}\right)\left(1-sin\varphi \right)\frac{\gamma }{\text{2}}{\left(H+D\right)}^{\text{2}}\\ +k_{\text{n}}k_{\text{a}}\frac{\gamma {\left[\left(b+b_{\text{0}}\right)tan\theta +h_{\text{0}}+h_{j}\right]}^{\text{2}}-\gamma {\left[\left(b+b_{\text{0}}\right)tan\theta +h_{\text{0}}\right]}^{\text{2}}+\gamma_{{\text{D}}_{\text{1}}}\left[{\left(h_{\text{0}}+h_{s}\right)}^{\text{2}}-\overset{\text{2}}{\underset{s}{h}}\right]-\gamma \overset{\text{2}}{\underset{s}{h}}}{2\left(a-1\right)}\\ +k_{\text{n}}k_{\text{a}}\frac{m_{\text{5}}{\left[\left(b+b_{\text{0}}\right)tan\theta +h_{\text{0}}+h_{j}\right]}^{1+a}-m_{\text{5}}{\left[\left(b+b_{\text{0}}\right)tan\theta +h_{\text{0}}\right]}^{1+a}}{a+1}\\ +\frac{m_{\text{8}}\left[{\left(h_{\text{0}}+h_{s}\right)}^{a+1}-\overset{a+1}{\underset{s}{h}}\right]+m_{\text{9}}\overset{a+1}{\underset{s}{h}}}{a+1} \end{array} \end{array}$$ (17)

## 3. Verification of the proposed calculation method

### 3.1 Laboratory test verification

#### 3.1.1 Test device.

Section 3 aims to verify the correctness and rationality of the derived analytical formulas for non-limit active earth pressure in finite soil masses under various failure modes. The test setup and basic parameters are based on the previous research findings of our research group to ensure data comparability [23].

**Fig 3:**
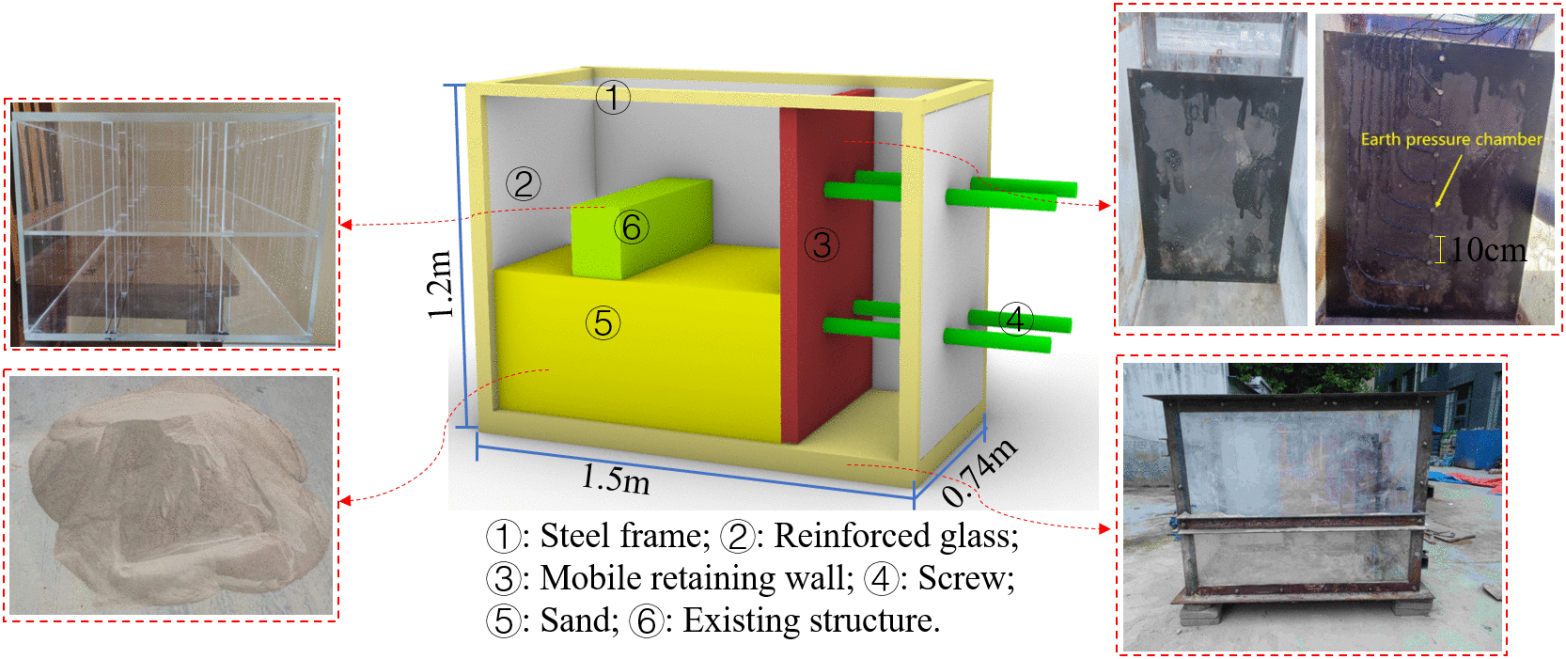
Test model box.

The model tank features a top-open rectangular casing, constructed through the assembly and welding of various components, including tempered glass side panels, a steel base plate, reinforced steel frame ring beams, fastening bolts, and angle steels. A movable steel plate is installed inside the model tank to simulate the translational retaining wall. Ten pressure cells with a measuring range of 200 kPa are arranged along the centerline of the movable retaining wall. The topmost and bottommost pressure cells are 5 cm away from the top and bottom of the retaining wall respectively, and the spacing between the rest is 10 cm. The dimensions of the model tank and retaining wall and the layout of pressure cells are presented in Fig 3.

#### 3.1.2 Test materials.

Natural air-dried fine sand was selected as the test fill material. A series of geotechnical laboratory tests were carried out to determine its physical and mechanical properties: the dry density of the sand is *ρ* = 1.51 g ⋅ cm^−3^, the internal friction angle is *φ* = 30°, and the cohesion is *c* = 0 kPa, which were measured by direct shear tests with a shear rate of 0.8 mm/min. The sieve analysis showed that the median particle size of the fine sand was approximately *d*_50_ = 0.4 mm. Since the thickness of the rigid retaining wall was 20 mm, about 50 times *d*_50_, the particle size effect could be neglected according to previous studies [24].

#### 3.1.3 Test scheme and process.

The test sand was placed using a layer-by-layer filling and compaction method. The thickness of each filling layer was fixed at 5 cm, and the mass of sand in each layer was controlled according to the sand density of *ρ* = 1.51 g ⋅ cm^−3^. Finally, the sand was compacted to the predetermined height. Subsequent to soil compaction, the existing structure model was positioned at the designated location, and monitoring sensors were installed as required. Throughout the test, soil pressure data were recorded after each 0.5 mm incremental displacement of the retaining wall. The test was terminated when the readings from the soil pressure cells stabilized (indicating the backfill soil had reached the active limit state). These procedures were replicated to complete a total of 8 test groups, with the detailed test parameters presented in Table 1.

**Table 1:** Test conditions.

Working condition	Proximity distance (*b*)/mm	Overburden thickness (*h*_j_)/mm	Notes
1	150	100	--
2	250	100	Standard working condition
3	350	100	--
4	450	100	--
5	250	200	--
6	250	300	--
7	250	400	--
8	--	--	Without existing structures

#### 3.1.4 Test results.

Laboratory tests were conducted under various working conditions, and the displacement values (*s*) corresponding to the limit state for each condition were extracted, as shown in Table 2. Based on the application conditions of soil failure modes, Working Condition 8 is classified under soil failure mode 1, while Working Conditions 4 and 7 are categorized as soil failure mode 2. Working Conditions 2, 3, 5, and 6 fall under soil failure mode 3, and Working Condition 1 is associated with soil failure mode 4.

**Table 2:** Results of laboratory tests.

Working condition	*H*/mm	*s*/mm	*s*/*H* × 1000	Failure mode
1	1000	2.5	2.5	Mode 4
2	1000	3.0	3.0	Mode 3
3	1000	3.0	3.0	Mode 3
4	1000	3.0	3.0	Mode 2
5	1000	3.0	3.0	Mode 3
6	1000	3.0	3.0	Mode 3
7	1000	3.0	3.0	Mode 2
8	1000	3.5	3.5	Mode 1

**Fig 4:**
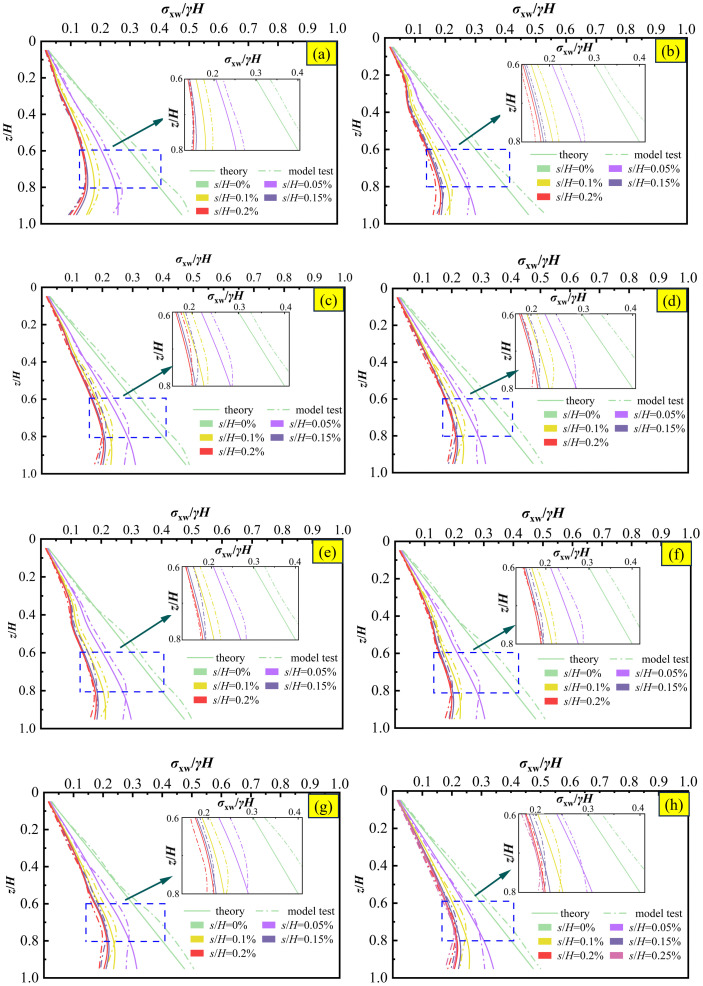
Comparison diagrams of different working conditions between laboratory tests and theoretical analyses: (a) Working condition 1; (b) Working condition 2; (c) Working condition 3; (d) Working condition 4; (e) Working condition 5; (f) Working condition 6; (g) Working condition 7; (h) Working condition 8.

A comparative study was conducted between the measured soil pressure values from the tests and the values obtained from theoretical calculation formulas, with the results presented in Fig 4. It can be observed from Fig 4 that, regardless of the working conditions, both the soil pressures obtained through the theoretical method and the laboratory tests exhibit non-linear curves. The laboratory test results are slightly higher than the theoretical calculated values, with most relative errors controlled within 10%, which is within a reasonable range and verifies the rationality of the theoretical calculation method.

### 3.2 Numerical simulation verification

#### 3.2.1 Numerical modeling and parameter selection.

**Fig 5:**
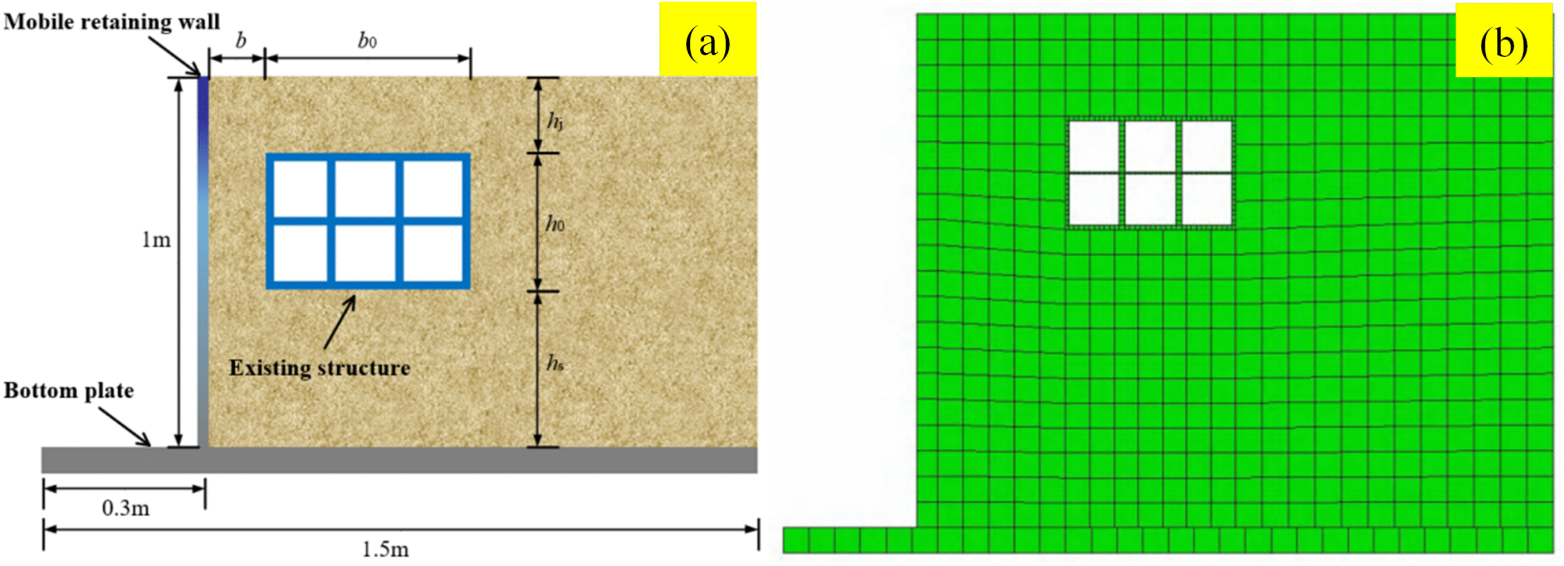
Schematic diagram. **(a)** The schematic diagram of the model; **(b)** The finite element numerical model.

To further validate the derived analytical formulas, two sets of finite element numerical simulations were conducted to investigate the distribution characteristics of non-limit active earth pressure under the translational displacement mode of retaining walls. The schematic diagram of the numerical model is shown in Fig 5(a). The retaining wall height is set to 1.0 m, and the width (*b*_*0*_) and height (*h*_0_) of the existing structure are 0.33 m and 0.216 m, respectively. Here, *h*_*j*_ denotes the overburden depth of the existing structure, and *b* represents the horizontal proximity distance between the foundation pit and the existing structure. The soil domain was discretized using quadrilateral elements under the 2D plane strain assumption, with a mesh size of 0.05 m. The final finite element model consists of 684 elements, as shown in Fig 5(b). The soil–structure interaction was simulated using contact elements. Vertical contact was adopted in the normal direction, while the penalty function was used in the tangential direction to describe interface friction. The friction coefficient of the soil–structure interfaces was set as tan*φ*2/3.

The Mohr-Coulomb constitutive model was adopted, and the soil parameters were set to match those used in the model test, as shown in Table 3.

**Table 3:** Soil parameter values for numerical simulation.

Parameter	*ρ*/(g·cm^-3^)	*φ*/°	*c*/kPa	*E*/MPa	*υ*	*ψ*/°
Value	1.51	30	0	30	0.43	0.1

#### 3.2.2 Numerical calculation conditions.

Two groups of calculation cases were selected to verify the derived theoretical formulas: (1) a proximity distance *b* = 0.3m with an overburden thickness *h*_*j*_ = 0.1m; and (2) a proximity distance *b* = 0.5m with an overburden thickness *h*_*j*_ = 0.1m. Case (1) corresponds to soil failure mode 3, while case (2) corresponds to soil failure mode 2.

#### 3.2.3 Numerical calculation results.

**Fig 6:**
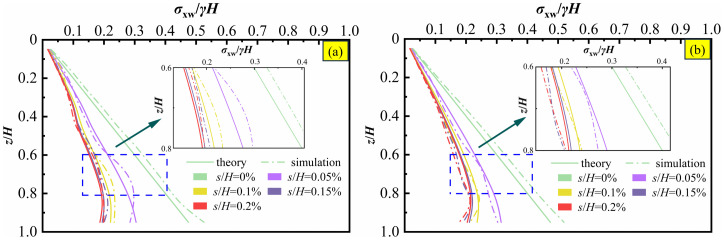
Comparison diagram between numerical simulation and theoretical calculation: (a) Working condition 1; (b) Working condition 2.

The comparison between the numerical simulation results and the theoretical calculation results is presented in Fig 6. It can be clearly seen from the figure that the non-limit active earth pressure distributions obtained from both methods are nonlinear curves under both working conditions. The relative error between the numerical and theoretical results is less than 5%, which further confirms the validity and accuracy of the proposed theoretical calculation method.

## 4. Non-limit active earth pressure isogram map

### 4.1 Concept of isogram map

The isogram of non-limit active earth pressure for limited soil mass is a diagram that connects points with the same ratio of non-limit active earth pressure to static earth pressure at various positions of existing underground structures. The specific implementation steps are as follows: (1) calculate the resultant force of earth pressure for traditional semi-infinite soil mass; (2) calculate the resultant force of non-limit active earth pressure for limited soil mass at any spatial position between existing underground structures and a new foundation pit; (3) determine the ratio of the resultant force of non-limit active earth pressure for limited soil mass to that of traditional semi-infinite soil mass at any spatial position between existing underground structures and a new foundation pit; (4) connect lines with the same ratio to form the isogram of non-limit active earth pressure for limited soil mass.

The isogram effectively represents the resultant force of non-limit earth pressure acting on the retaining structure of the foundation pit at any spatial position between existing underground structures and the new foundation pit. Additionally, it establishes the relationship between the resultant force of non-limit active earth pressure of the limited soil mass and that of the semi-infinite soil mass.

### 4.2 Isogram map calculation process

**Fig 7:**
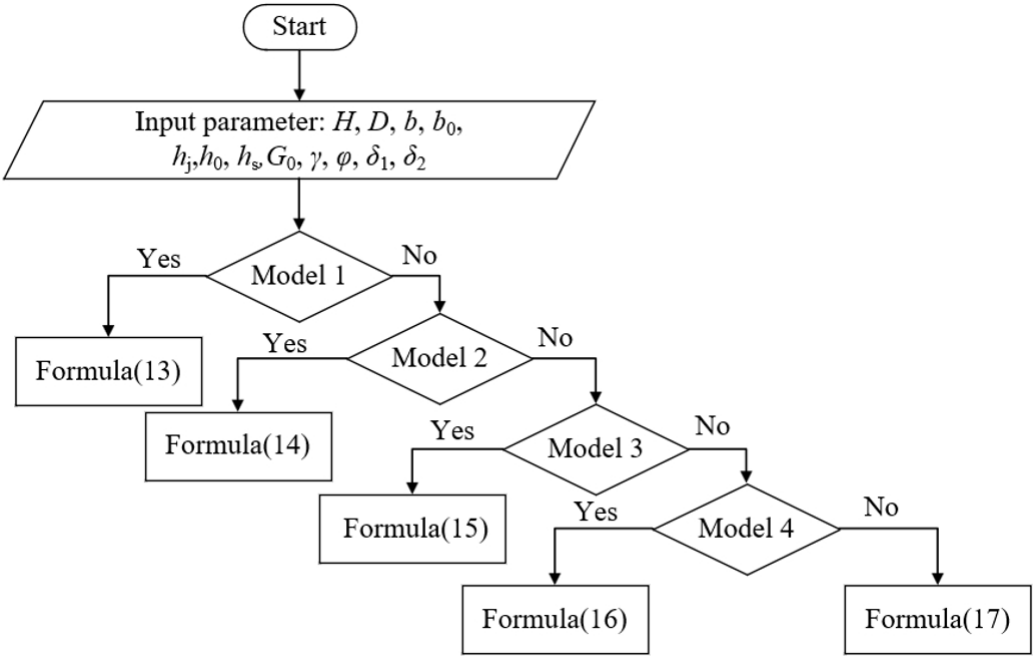
Flow chart of earth pressure calculation program.

Based on the earth pressure formulas derived in Section 2, the calculation flow chart of non-limit active earth pressure for limited soil masses is established, as shown in Fig 7. Using this calculation process, the non-limit active earth pressure intensity and resultant force acting on the retaining structure can be obtained for any combination of engineering parameters. By calculating the ratio of the limited soil earth pressure resultant force to the semi-infinite soil earth pressure resultant force at different spatial positions and connecting points with the same ratio, the non-limit active earth pressure isogram can be generated.

### 4.3 Example analysis

**Fig 8:**
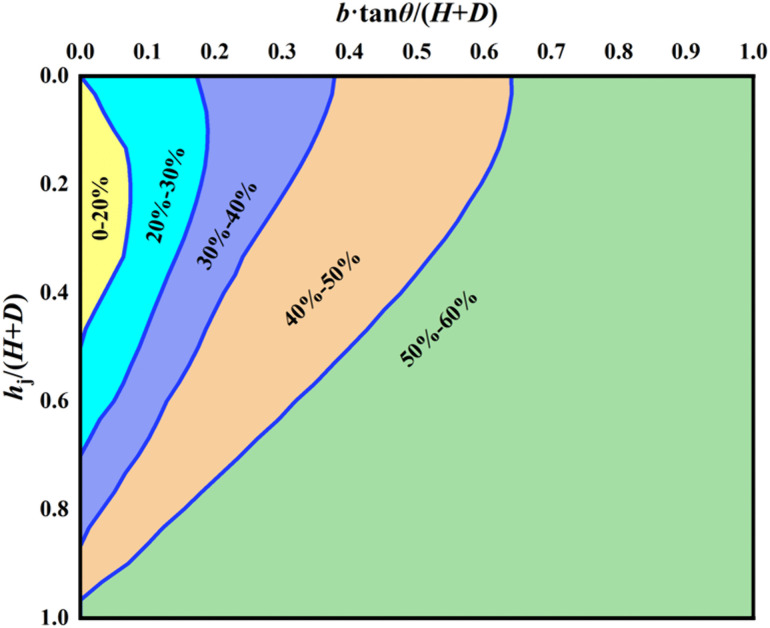
Isogram of non-limit limited earth pressure.

An example calculation is provided with the following parameters: no surcharge on the ground, *γ* = 16.2 kN/m³, *c* = 0 kPa, *φ* = 35°, *δ*_1_ = *δ*_2_ = 20°, *H* = 25 m, *D* = 5 m, *h*_0_ = 12.4 m, and *b*_0_ = 21.2 m. Utilizing the proposed theoretical calculation method for non-limit active earth pressure, a soil displacement of *u* = 0.2 mm is considered. The isogram lines are generated by the ratio of the resultant force of non-limit active earth pressure for a limited soil mass on the retaining structure to that of traditional semi-infinite soil mass under multiple working conditions (specifically, the proximity distance *b* and overburden depth *h*_*j*_ of existing underground structures), as illustrated in Fig 8. It is evident from Fig 8 that the isolines exhibit a non-linear distribution. As the proximity distance and overburden depth of existing underground structures increase, the resultant force of non-limit active earth pressure gradually rises, resulting in a sparser distribution of isolines, with the maximum value not exceeding 0.6*E*_na_.

#### 4.3.1 Overburden depth.

**Fig 9:**
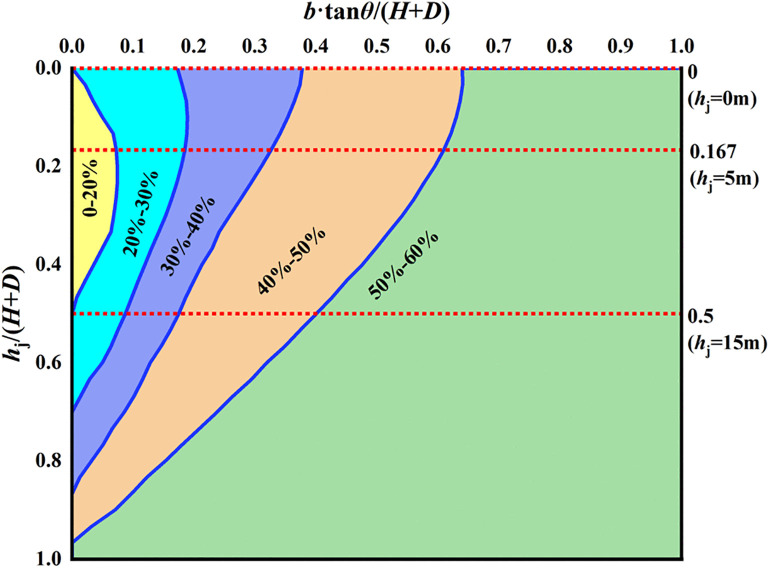
Distribution diagram of earth pressure under different proximity distances.

When the soil displacement is 0.2 mm, the resultant forces of limited soil earth pressure for overburden depths of existing underground structures at 0 m, 5 m, and 15 m are illustrated as red lines in Fig 9. The red line corresponding to *h*_*j*_ = 0 m represents the resultant forces of limited soil earth pressure at varying proximity distances when the overburden depth of the existing underground structures is 0 m.

As illustrated in Fig 9, under the same overburden depth of existing underground structures, the non-limit earth pressure exerted by the surrounding soil on the retaining structure gradually increases with the increase in proximity distance. Furthermore, the larger the spacing of isolines, the more the sensitivity of earth pressure diminishes with increasing proximity distance. As the overburden depth increases, the red line intersects fewer isoline intervals, indicating that a greater overburden depth results in a weaker influence of proximity distance on earth pressure.

#### 4.3.2 Proximity distance.

**Fig 10:**
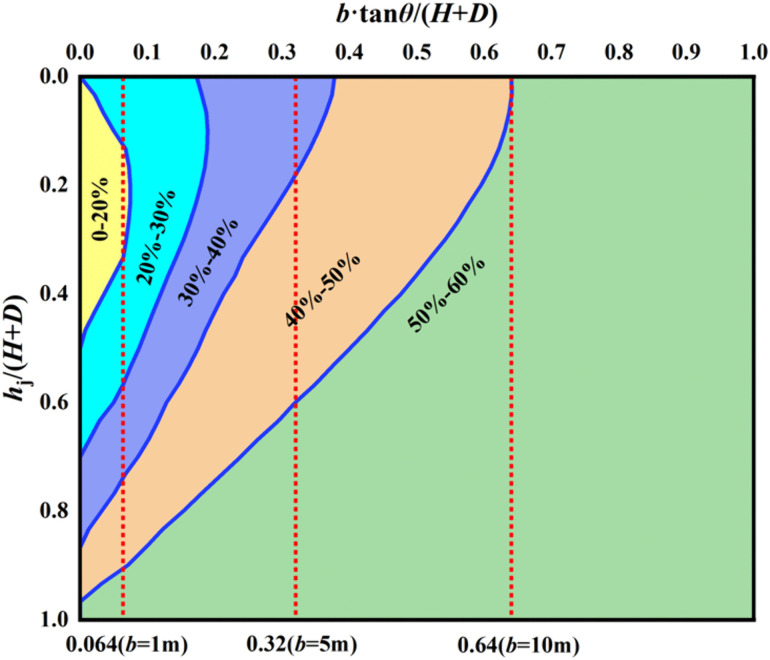
Distribution diagram of earth pressure under different overburden depths.

When the proximity distances are 1 m, 5 m, and 10 m, the resultant forces of limited soil earth pressure are depicted by the red lines in Fig 10. The red line corresponding to *b* = 1 m illustrates the resultant forces of limited soil earth pressure under varying burial depths of existing underground structures for a proximity distance of 1 m.

It can be observed from Fig 10 that when the horizontal distance between the existing underground structure and the foundation pit is less than 1 m, the non-limit earth pressure first decreases and then increases with increasing overburden depth. In contrast, when the horizontal distance exceeds 1 m, the non-limit earth pressure increases monotonically with overburden depth. As the proximity distance increases, the red line intersects fewer isogram intervals, indicating that a larger proximity distance reduces the influence of overburden depth on earth pressure.

### 4.4 Parameter influence analysis

#### 4.4.1 Soil displacement.

**Fig 11:**
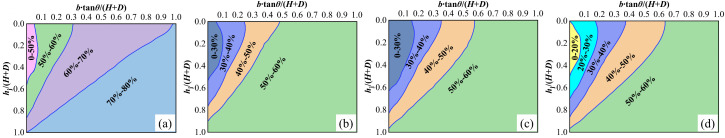
Influence diagram of soil displacement on non-limit active earth pressure: (a)*u* = 0.05 mm; (b)*u* = 0.1 mm; (c)*u* = 0.15 mm; (d)*u* = 0.2 mm.

The influence of soil displacement on the non-limit active earth pressure resultant force of limited soil mass was analyzed by considering four displacement levels: 0.05 mm, 0.1 mm, 0.15 mm and 0.2 mm, with all other parameters kept constant. The results are shown in Fig 11.

As illustrated in Fig 11, the non-limit active earth pressure resultant force decreases monotonically with increasing soil displacement, which is consistent with the fundamental principle that active earth pressure decreases as retaining wall displacement increases. Notably, the earth pressure decreases rapidly when the soil displacement increases from 0.05 mm to 0.1 mm, while the decreasing rate slows down significantly when the displacement increases from 0.1 mm to 0.2 mm. This indicates that the earth pressure is more sensitive to small displacements in the initial stage of retaining wall movement.

#### 4.4.2 Foundation pit depth.

**Fig 12:**
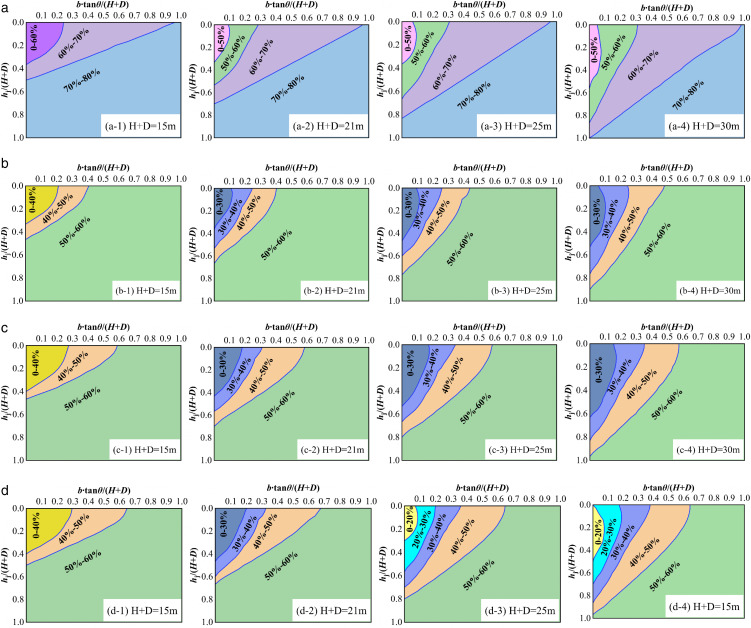
Influence diagram of foundation pit depth on non-limit earth pressure under different soil displacements: (a) *u* = 0.05 mm; (b) *u* = 0.1 mm; (c) *u* = 0.15 mm; (d) *u* = 0.2 mm.

By changing the foundation pit depth and maintaining the insertion ratio at 5:1, four foundation pit depths (*H*) are selected: 12.5 m, 17.5 m, 20.8 m, and 25 m, with corresponding embedded depths (*D*) of 2.5 m, 3.5 m, 4.2 m, and 5 m. *H* + *D* are 15 m, 21 m, 25 m, and 30 m respectively. For each foundation pit depth, the resultant forces of non-limit active earth pressure under soil displacements of *u* = 0.05 mm, 0.1 mm, 0.15 mm, and 0.2 mm are calculated to analyze the influence of foundation pit depth on the resultant force of non-limit earth pressure. The results are shown in Fig 12.

As illustrated in Fig 12, under identical soil displacement conditions, the resultant force of the non-limit active earth pressure of the confined soil mass gradually decreases with increasing foundation pit depth. As the length of the retaining structure increases, the initiation point of the slip surface progressively shifts downward, causing the starting points of each isoline to converge downward.

#### 4.4.3 Soil internal friction angle.

**Fig 13:**
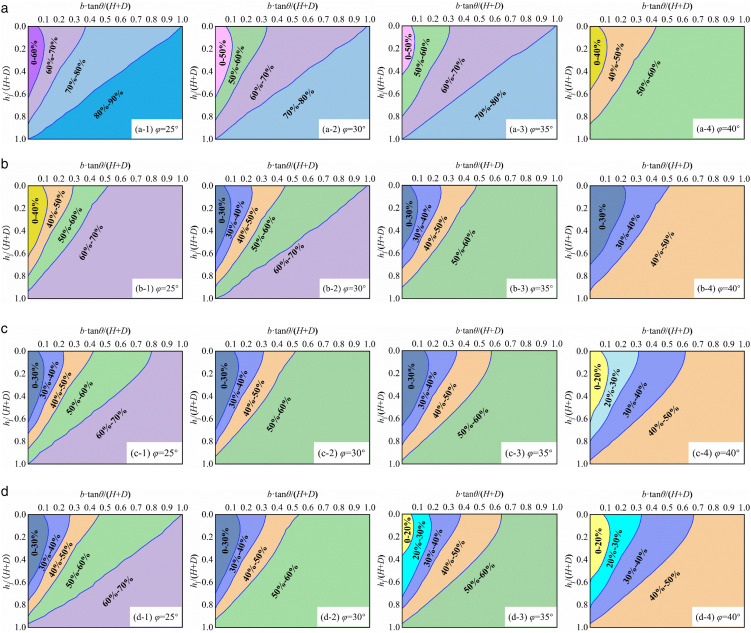
Influence diagram of internal friction angle on non-limit active earth pressure under different soil displacements: (a) *u* = 0.05 mm; (b)*u* = 0.1 mm; (c) *u* = 0.15 mm; (d) *u* = 0.2 mm.

The internal friction angles are selected as 25°, 30°, 35°, and 40°, and the wall-soil friction angles are selected according to the ratio of (*δ*_1_/*φ* = *δ*_2_/*φ* = 2/3). For each internal friction angle, the resultant forces of earth pressure under four soil displacements of *u* = 0.05 mm, 0.1 mm, 0.15 mm, and 0.2 mm are calculated with other parameters unchanged to analyze the influence of internal friction angle on the resultant force of non-limit active earth pressure of limited soil mass. The results are shown in Fig 13.

As illustrated in Fig 13, under identical soil displacement conditions, the resultant force of non-limit active earth pressure in a limited soil mass gradually decreases as the internal friction angle of the soil increases. When the friction angle (*φ*) is between 25° and 35°, it has a minimal impact on the resultant force of limited soil earth pressure. However, when *φ* ranges from 35° to 40°, the internal friction angle significantly influences the resultant force of limited soil earth pressure.

#### 4.4.4 Wall-soil friction angle.

**Fig 14:**
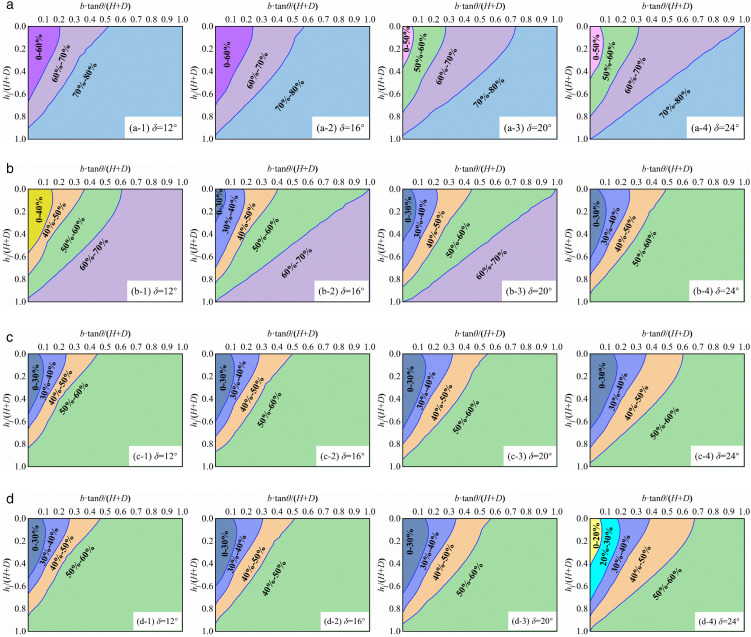
Influence diagram of wall-soil friction angle on non-limit active earth pressure under different soil displacements: (a) *u* = 0.05 mm; (b)*u* = 0.1 mm; (c)*u* = 0.15 mm; (d)*u* = 0.2 mm.

The wall-soil friction angles are selected as 12°, 16°, 20°, and 24°, with other parameters unchanged. The resultant forces of earth pressure under four soil displacements of *u* = 0.05 mm, 0.1 mm, 0.15 mm, and 0.2 mm are calculated to analyze the influence of wall-soil friction angle on the resultant force of non-limit active earth pressure of limited soil mass. The results are shown in Fig 14. As can be seen from Fig 14, regardless of the soil displacement, the resultant force of non-limit active earth pressure of limited soil mass gradually decreases with the increase of wall-soil friction angle. Compared with the internal friction angle of soil, the resultant force of earth pressure decreases more slowly.

## 5. Simple calculation method for non-limit active earth pressure

By integrating the static earth pressure intensity formula, the corresponding resultant force formula of static earth pressure can be obtained, as shown in equation (18).


$$\begin{array}{c} E_{\text{0}}=\int_{\text{0}}^{H+D}p_{\text{0}}\text{d}z=\left(1-sin\varphi \right)\frac{\gamma }{\text{2}}{\left(H+D\right)}^{\text{2}} \end{array}$$ (18)

To simplify the calculation formula for the resultant force of non-limit active earth pressure in a limited soil mass, a spatial position relationship coefficient is multiplied by the resultant force of static earth pressure. This results in a proposed simplified calculation method for the resultant force of non-limit active earth pressure in limited soil mass, as demonstrated in equation (19).


$$\begin{array}{c} E_{\text{na}}=\lambda \cdot E_{\text{0}} \end{array}$$ (19)

In the formula, *λ* is the spatial position relationship coefficient of the resultant force of active earth pressure of limited soil mass.

**Fig 15:**
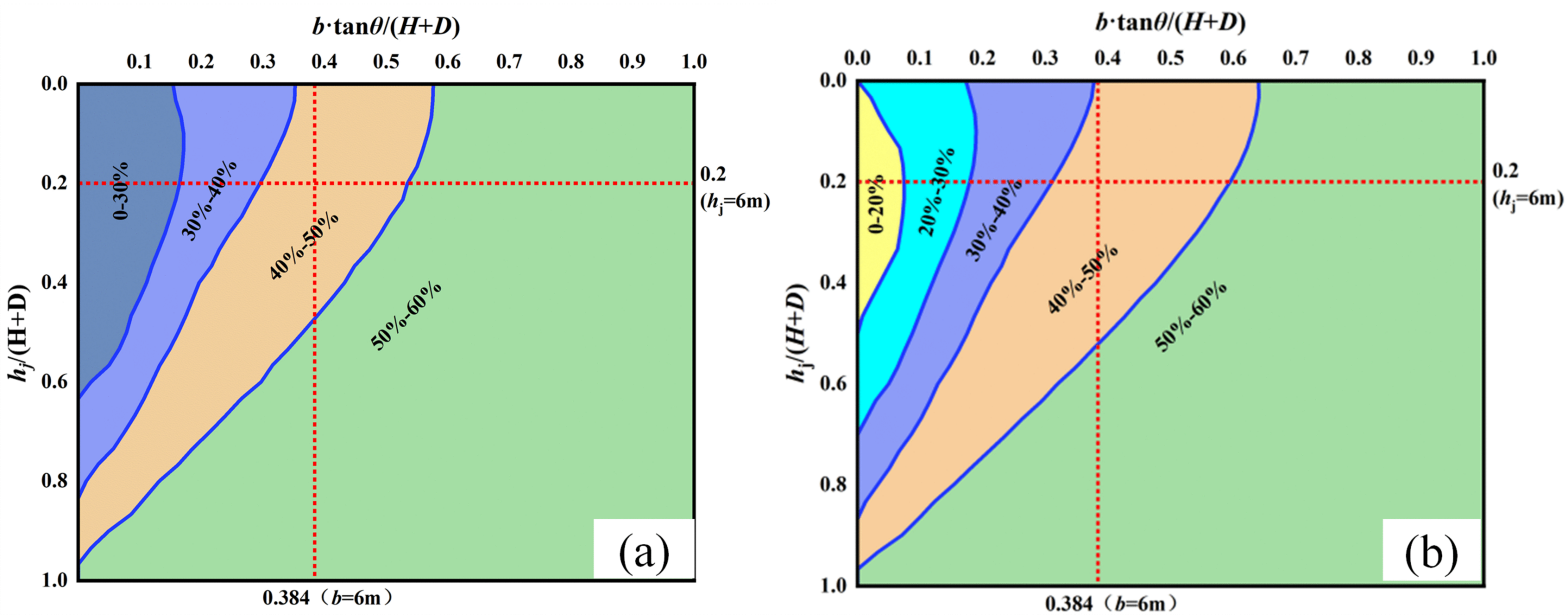
Recommended value diagram of *λ*: (a)*u* = 0.15%*u*max; (b)*u* = 0.2%*u*max.

Taking the foundation pit retaining structure of 30 m (*H* + *D* = 30 m), proximity distance *b* = 6 m, overburden depth of existing metro station *h*_j_ = 6 m, and soil displacements *u* of 0.15%*u*_max_ and 0.2%*u*_max_ as examples, the derivation of the recommended value of *λ* is given, and the calculation results are shown in Fig 15. As can be seen from Fig 15, the resultant force of earth pressure on the finite soil mass under the above conditions ranges from 40% to 50%. Accordingly, the upper limit of this interval is adopted, and *λ* = 0.5.

According to the above principles, by changing the parameters of proximity distance and overburden depth, 120 cases were set up for *u* = 0.05%*u*_max_, 0.1%*u*_max_, 0.15%*u*_max_, and 0.2%*u*_max_ under *H* + *D* = 15 m, totaling 480 cases. Under *H* + *D* = 21 m, 231 cases were set up for the same soil displacements, totaling 924 cases. Under *H* + *D* = 25 m, 325 cases were set up, totaling 1300 cases. Under *H* + *D* = 30 m, 480 cases were set up, totaling 1920 cases. Through numerical calculations for the above cases, the spatial position relationship coefficients (*λ*) of the resultant force of active earth pressure of limited soil mass are summarized in Tables 4–19. Among them, *u* = 0.05%*u*_max_, 0.1%*u*_max_, and 0.15%*u*_max_ represent non-limit earth pressure, while *u* = 0.2%*u*_max_ represents limit earth pressure.

**Table 4:** Recommended value of *λ* for *u* = 0.05%*u*_max_ and *H* + *D* = 15 m.

Working condition	*b*(m)							
	1	2	3	4	5	6	7	≥8
*h*_j_=3m	0.6	0.7	0.7	0.7	0.7	0.7	0.8	0.8
*h*_j_=6m	0.6	0.7	0.7	0.7	0.8	0.8	0.8	0.8
*h*_j_=9m	0.7	0.7	0.7	0.8	0.8	0.8	0.8	0.8
*h*_j_=12m	0.7	0.8	0.8	0.8	0.8	0.8	0.8	0.8
*h*_j_ ≥ 15m	0.8	0.8	0.8	0.8	0.8	0.8	0.8	0.8

**Table 5:** Recommended value of *λ* for *u* = 0.1%*u*_max_ and *H* + *D* = 15 m.

Working condition	*b*(m)							
	1	2	3	4	5	6	7	≥8
*h*_j_=3m	0.4	0.5	0.6	0.6	0.6	0.6	0.6	0.6
*h*_j_=6m	0.4	0.5	0.6	0.6	0.6	0.6	0.6	0.6
*h*_j_=9m	0.5	0.6	0.6	0.6	0.6	0.6	0.6	0.6
*h*_j_=12m	0.6	0.6	0.6	0.6	0.6	0.6	0.6	0.6
*h*_j_ ≥ 15m	0.6	0.6	0.6	0.6	0.6	0.6	0.6	0.6

**Table 6:** Recommended value of *λ* for *u* = 0.15%*u*_max_ and *H* + *D* = 15 m.

Working condition	*b*(m)							
	1	2	3	4	5	6	7	≥8
*h*_j_=3m	0.3	0.5	0.5	0.5	0.6	0.6	0.6	0.6
*h*_j_=6m	0.4	0.5	0.5	0.6	0.6	0.6	0.6	0.6
*h*_j_=9m	0.5	0.5	0.6	0.6	0.6	0.6	0.6	0.6
*h*_j_=12m	0.5	0.6	0.6	0.6	0.6	0.6	0.6	0.6
*h*_j_ ≥ 15m	0.6	0.6	0.6	0.6	0.6	0.6	0.6	0.6

**Table 7:** Recommended value of *λ* for *u* = 0.2%*u*_max_ and *H* + *D* = 15 m.

Working condition	*b*(m)							
	1	2	3	4	5	6	7	≥8
*h*_j_=3m	0.3	0.4	0.5	0.5	0.6	0.6	0.6	0.6
*h*_j_=6m	0.4	0.5	0.5	0.6	0.6	0.6	0.6	0.6
*h*_j_=9m	0.4	0.5	0.6	0.6	0.6	0.6	0.6	0.6
*h*_j_=12m	0.5	0.6	0.6	0.6	0.6	0.6	0.6	0.6
*h*_j_ ≥ 15m	0.6	0.6	0.6	0.6	0.6	0.6	0.6	0.6

**Table 8:** Recommended value of *λ* for *u* = 0.05%*u*_max_ and *H* + *D* = 21 m.

Working condition	*b*(m)										
	1	2	3	4	5	6	7	8	9	10	≥11
*h*_j_=3m	0.5	0.6	0.7	0.7	0.7	0.7	0.7	0.7	0.7	0.8	0.8
*h*_j_=6m	0.6	0.6	0.7	0.7	0.7	0.7	0.7	0.8	0.8	0.8	0.8
*h*_j_=9m	0.6	0.7	0.7	0.7	0.7	0.7	0.8	0.8	0.8	0.8	0.8
*h*_j_=12m	0.6	0.7	0.7	0.7	0.8	0.8	0.8	0.8	0.8	0.8	0.8
*h*_j_=15m	0.7	0.7	0.7	0.8	0.8	0.8	0.8	0.8	0.8	0.8	0.8
*h*_j_=18m	0.7	0.8	0.8	0.8	0.8	0.8	0.8	0.8	0.8	0.8	0.8
*h*_j_ ≥ 21m	0.8	0.8	0.8	0.8	0.8	0.8	0.8	0.8	0.8	0.8	0.8

**Table 9:** Recommended value of *λ* for *u* = 0.1%*u*_max_ and *H* + *D* = 21 m.

Working condition	*b*(m)										
	1	2	3	4	5	6	7	8	9	10	≥11
*h*_j_=3m	0.3	0.4	0.5	0.5	0.6	0.6	0.6	0.6	0.6	0.6	0.6
*h*_j_=6m	0.4	0.5	0.5	0.6	0.6	0.6	0.6	0.6	0.6	0.6	0.6
*h*_j_=9m	0.4	0.5	0.5	0.6	0.6	0.6	0.6	0.6	0.6	0.6	0.6
*h*_j_=12m	0.5	0.5	0.6	0.6	0.6	0.6	0.6	0.6	0.6	0.6	0.6
*h*_j_=15m	0.5	0.6	0.6	0.6	0.6	0.6	0.6	0.6	0.6	0.6	0.6
*h*_j_=18m	0.6	0.6	0.6	0.6	0.6	0.6	0.6	0.6	0.6	0.6	0.6
*h*_j_ ≥ 21m	0.6	0.6	0.6	0.6	0.6	0.6	0.6	0.6	0.6	0.6	0.6

**Table 10:** Recommended value of *λ* for *u* = 0.15%*u*_max_ and *H* + *D* = 21 m.

Working condition	*b*(m)										
	1	2	3	4	5	6	7	8	9	10	≥11
*h*_j_=3m	0.3	0.4	0.5	0.5	0.5	0.5	0.6	0.6	0.6	0.6	0.6
*h*_j_=6m	0.3	0.4	0.5	0.5	0.5	0.6	0.6	0.6	0.6	0.6	0.6
*h*_j_=9m	0.3	0.4	0.5	0.5	0.6	0.6	0.6	0.6	0.6	0.6	0.6
*h*_j_=12m	0.4	0.5	0.5	0.6	0.6	0.6	0.6	0.6	0.6	0.6	0.6
*h*_j_=15m	0.5	0.5	0.6	0.6	0.6	0.6	0.6	0.6	0.6	0.6	0.6
*h*_j_=18m	0.6	0.6	0.6	0.6	0.6	0.6	0.6	0.6	0.6	0.6	0.6
*h*_j_ ≥ 21m	0.6	0.6	0.6	0.6	0.6	0.6	0.6	0.6	0.6	0.6	0.6

**Table 11:** Recommended value of *λ* for *u* = 0.2%*u*_max_ and *H* + *D* = 21 m.

Working condition	*b*(m)										
	1	2	3	4	5	6	7	8	9	10	≥11
*h*_j_=3m	0.3	0.3	0.4	0.5	0.5	0.5	0.5	0.6	0.6	0.6	0.6
*h*_j_=6m	0.3	0.4	0.5	0.5	0.5	0.6	0.6	0.6	0.6	0.6	0.6
*h*_j_=9m	0.3	0.4	0.5	0.5	0.6	0.6	0.6	0.6	0.6	0.6	0.6
*h*_j_=12m	0.4	0.5	0.5	0.6	0.6	0.6	0.6	0.6	0.6	0.6	0.6
*h*_j_=15m	0.5	0.5	0.6	0.6	0.6	0.6	0.6	0.6	0.6	0.6	0.6
*h*_j_=18m	0.5	0.6	0.6	0.6	0.6	0.6	0.6	0.6	0.6	0.6	0.6
*h*_j_ ≥ 21m	0.6	0.6	0.6	0.6	0.6	0.6	0.6	0.6	0.6	0.6	0.6

**Table 12:** Recommended value of *λ* for *u* = 0.05%*u*_max_ and *H* + *D* = 25 m.

Working condition	*b*(m)						
	1	3	5	7	9	11	≥13
*h*_j_=3m	0.5	0.6	0.7	0.7	0.7	0.7	0.8
*h*_j_=6m	0.5	0.6	0.7	0.7	0.7	0.8	0.8
*h*_j_=9m	0.6	0.6	0.7	0.7	0.8	0.8	0.8
*h*_j_=12m	0.6	0.7	0.7	0.8	0.8	0.8	0.8
*h*_j_=15m	0.6	0.7	0.7	0.8	0.8	0.8	0.8
*h*_j_=18m	0.7	0.7	0.8	0.8	0.8	0.8	0.8
*h*_j_=21m	0.7	0.8	0.8	0.8	0.8	0.8	0.8
*h*_j_ ≥ 24m	0.8	0.8	0.8	0.8	0.8	0.8	0.8

**Table 13:** Recommended value of *λ* for *u* = 0.1%*u*_max_ and *H* + *D* = 25 m.

Working condition	*b*(m)						
	1	3	5	7	9	11	≥13
*h*_j_=3m	0.3	0.4	0.5	0.6	0.6	0.6	0.6
*h*_j_=6m	0.3	0.5	0.6	0.6	0.6	0.6	0.6
*h*_j_=9m	0.4	0.5	0.6	0.6	0.6	0.6	0.6
*h*_j_=12m	0.4	0.5	0.6	0.6	0.6	0.6	0.6
*h*_j_=15m	0.5	0.6	0.6	0.6	0.6	0.6	0.6
*h*_j_=18m	0.5	0.6	0.6	0.6	0.6	0.6	0.6
*h*_j_=21m	0.6	0.6	0.6	0.6	0.6	0.6	0.6
*h*_j_ ≥ 24m	0.6	0.6	0.6	0.6	0.6	0.6	0.6

**Table 14:** Recommended value of *λ* for *u* = 0.15%*u*_max_ and *H* + *D* = 25 m.

Working condition	*b*(m)						
	1	3	5	7	9	11	≥13
*h*_j_=3m	0.3	0.4	0.5	0.5	0.6	0.6	0.6
*h*_j_=6m	0.3	0.4	0.5	0.6	0.6	0.6	0.6
*h*_j_=9m	0.3	0.5	0.5	0.6	0.6	0.6	0.6
*h*_j_=12m	0.3	0.5	0.6	0.6	0.6	0.6	0.6
*h*_j_=15m	0.4	0.5	0.6	0.6	0.6	0.6	0.6
*h*_j_=18m	0.5	0.6	0.6	0.6	0.6	0.6	0.6
*h*_j_=21m	0.5	0.6	0.6	0.6	0.6	0.6	0.6
*h*_j_ ≥ 24m	0.6	0.6	0.6	0.6	0.6	0.6	0.6

**Table 15:** Recommended value of *λ* for *u* = 0.2%*u*_max_ and *H* + *D* = 25 m.

Working condition	*b*(m)						
	1	3	5	7	9	11	≥13
*h*_j_=3m	0.2	0.4	0.5	0.5	0.6	0.6	0.6
*h*_j_=6m	0.2	0.4	0.5	0.5	0.6	0.6	0.6
*h*_j_=9m	0.3	0.5	0.5	0.6	0.6	0.6	0.6
*h*_j_=12m	0.3	0.5	0.5	0.6	0.6	0.6	0.6
*h*_j_=15m	0.4	0.5	0.6	0.6	0.6	0.6	0.6
*h*_j_=18m	0.5	0.6	0.6	0.6	0.6	0.6	0.6
*h*_j_=21m	0.5	0.6	0.6	0.6	0.6	0.6	0.6
*h*_j_ ≥ 24m	0.6	0.6	0.6	0.6	0.6	0.6	0.6

**Table 16:** Recommended value of *λ* for *u* = 0.05%*u*_max_ and *H* + *D* = 30 m.

Working condition	*b*(m)								
	1	3	5	7	9	11	13	15	≥17
*h*_j_=3m	0.5	0.6	0.7	0.7	0.7	0.7	0.7	0.8	0.8
*h*_j_=6m	0.5	0.6	0.7	0.7	0.7	0.7	0.8	0.8	0.8
*h*_j_=9m	0.5	0.6	0.7	0.7	0.7	0.8	0.8	0.8	0.8
*h*_j_=12m	0.6	0.7	0.7	0.7	0.7	0.8	0.8	0.8	0.8
*h*_j_=15m	0.6	0.7	0.7	0.7	0.8	0.8	0.8	0.8	0.8
*h*_j_=18m	0.6	0.7	0.7	0.8	0.8	0.8	0.8	0.8	0.8
*h*_j_=21m	0.7	0.7	0.8	0.8	0.8	0.8	0.8	0.8	0.8
*h*_j_=24m	0.7	0.7	0.8	0.8	0.8	0.8	0.8	0.8	0.8
*h*_j_=27m	0.7	0.8	0.8	0.8	0.8	0.8	0.8	0.8	0.8
*h*_j_ ≥ 30m	0.8	0.8	0.8	0.8	0.8	0.8	0.8	0.8	0.8

**Table 17:** Recommended value of *λ* for *u* = 0.1%*u*_max_ and *H* + *D* = 30 m.

Working condition	*b*(m)								
	1	3	5	7	9	11	13	15	≥17
*h*_j_=3m	0.3	0.4	0.5	0.6	0.6	0.6	0.6	0.6	0.6
*h*_j_=6m	0.3	0.4	0.5	0.6	0.6	0.6	0.6	0.6	0.6
*h*_j_=9m	0.3	0.5	0.5	0.6	0.6	0.6	0.6	0.6	0.6
*h*_j_=12m	0.3	0.5	0.6	0.6	0.6	0.6	0.6	0.6	0.6
*h*_j_=15m	0.4	0.5	0.6	0.6	0.6	0.6	0.6	0.6	0.6
*h*_j_=18m	0.4	0.5	0.6	0.6	0.6	0.6	0.6	0.6	0.6
*h*_j_=21m	0.5	0.6	0.6	0.6	0.6	0.6	0.6	0.6	0.6
*h*_j_=24m	0.5	0.6	0.6	0.6	0.6	0.6	0.6	0.6	0.6
*h*_j_=27m	0.6	0.6	0.6	0.6	0.6	0.6	0.6	0.6	0.6
*h*_j_ ≥ 30m	0.6	0.6	0.6	0.6	0.6	0.6	0.6	0.6	0.6

**Table 18:** Recommended value of *λ* for *u* = 0.15%*u*_max_ and *H* + *D* = 30 m.

Working condition	*b*(m)								
	1	3	5	7	9	11	13	15	≥17
*h*_j_=3m	0.3	0.4	0.4	0.5	0.6	0.6	0.6	0.6	0.6
*h*_j_=6m	0.3	0.4	0.5	0.5	0.6	0.6	0.6	0.6	0.6
*h*_j_=9m	0.3	0.4	0.5	0.5	0.6	0.6	0.6	0.6	0.6
*h*_j_=12m	0.3	0.4	0.5	0.6	0.6	0.6	0.6	0.6	0.6
*h*_j_=15m	0.3	0.5	0.5	0.6	0.6	0.6	0.6	0.6	0.6
*h*_j_=18m	0.4	0.5	0.6	0.6	0.6	0.6	0.6	0.6	0.6
*h*_j_=21m	0.4	0.5	0.6	0.6	0.6	0.6	0.6	0.6	0.6
*h*_j_=24m	0.5	0.6	0.6	0.6	0.6	0.6	0.6	0.6	0.6
*h*_j_=27m	0.6	0.6	0.6	0.6	0.6	0.6	0.6	0.6	0.6
*h*_j_ ≥ 30m	0.6	0.6	0.6	0.6	0.6	0.6	0.6	0.6	0.6

**Table 19:** Recommended value of *λ* for *u* = 0.2%*u*_max_ and *H* + *D* = 30 m.

Working condition	*b*(m)								
	1	3	5	7	9	11	13	15	≥17
*h*_j_=3m	0.3	0.4	0.4	0.5	0.5	0.6	0.6	0.6	0.6
*h*_j_=6m	0.2	0.4	0.5	0.5	0.5	0.6	0.6	0.6	0.6
*h*_j_=9m	0.2	0.4	0.5	0.5	0.6	0.6	0.6	0.6	0.6
*h*_j_=12m	0.3	0.4	0.5	0.5	0.6	0.6	0.6	0.6	0.6
*h*_j_=15m	0.3	0.5	0.5	0.6	0.6	0.6	0.6	0.6	0.6
*h*_j_=18m	0.4	0.5	0.6	0.6	0.6	0.6	0.6	0.6	0.6
*h*_j_=21m	0.4	0.5	0.6	0.6	0.6	0.6	0.6	0.6	0.6
*h*_j_=24m	0.5	0.6	0.6	0.6	0.6	0.6	0.6	0.6	0.6
*h*_j_=27m	0.6	0.6	0.6	0.6	0.6	0.6	0.6	0.6	0.6
*h*_j_ ≥ 30m	0.6	0.6	0.6	0.6	0.6	0.6	0.6	0.6	0.6

## 6. Conclusion

(1) For different failure modes of soil mass, the analytical expressions for the resultant force of non-limit active earth pressure of limited soil mass are derived, and the correctness and rationality of the calculation method are verified through laboratory tests and numerical simulations.
(2) The concept of an isoline diagram for the resultant force of non-limit active earth pressure in a limited soil mass is proposed, and its calculation process is established. The results show that the isoline diagrams generally exhibit a nonlinear and relatively sparse distribution. As the proximity distance increases, the sensitivity of earth pressure to proximity distance gradually decreases; meanwhile, with increasing overburden depth, the influence of proximity distance on earth pressure is weakened. The resultant force of non-limit active earth pressure decreases with increasing soil displacement and foundation pit depth. For the soil parameters, the soil internal friction angle has a significant influence on the resultant force when it ranges from 35° to 40°. For the interface parameters, the resultant force gradually decreases with increasing wall-soil friction angle, but the decreasing trend is relatively slow.
(3) A simple calculation method for earth pressure of limited soil mass in foundation pit engineering adjacent to existing underground structures is proposed, the spatial position relationship coefficient of the resultant force of active earth pressure of limited soil mass is given, and the recommended value of the spatial position relationship coefficient of the resultant force of active earth pressure of limited soil mass is provided in combination with the research content.
(4) This study mainly focuses on the non-limit active earth pressure of finite soil mass in homogeneous strata for foundation pit projects adjacent to existing structures. The proposed method is therefore mainly applicable to homogeneous soil conditions and translational displacement of retaining structures. However, the effects of complex geological conditions, groundwater, and other factors have not been fully considered in this study, and these issues will be further investigated in future research.
